# Supplementary material for: Coupling of organic and inorganic aerosol systems and the effect on gas–particle partitioning in the southeastern US
Source: Atmos Chem Phys. Author manuscript; Available in PMC 2018 Jun 27. (PMC6020690; doi:10.5194/acp-18-357-2018)
Supplement: Sup1 [file NIHMS963019-supplement-Sup1.pdf]

Supplement of Atmos. Chem. Phys., 18, 357–370, 2018
https://doi.org/10.5194/acp-18-357-2018-supplement
© Author(s) 2018. This work is distributed under
the Creative Commons Attribution 4.0 License.

Supplement of

Coupling of organic and inorganic aerosol systems and the effect on gas–particle partitioning in the southeastern US

Havala O. T. Pye et al.

Correspondence to: Havala O. T. Pye (pye.havala@epa.gov)

The copyright of individual parts of the supplement might differ from the CC BY 4.0 License.

Table S1: Functional group assignments of organic compounds and factors used as species in AIOMFAC. AIOMFAC does not include experimentally-constrained interaction parameters for the bisulfate anion with ester, aldehyde, ketone, or aromatic carbon-alcohol functional groups (Zuend and Seinfeld, 2012), although an analogy approach can be employed to estimate these interactions. In addition, organonitrate -- ion interaction parameters are not yet available. When needed, these functional groups were assigned to another representative group. Isoprene-OA used in AIOMFAC consisted of measured Isoprene-OA minus explicitly represented isoprene-derived compounds. LO-OOA used in AIOMFAC consisted of measured LO-OOA minus explicitly represented monoterpene-derived compounds. BBOA used in AIOMFAC consisted of measured BBOA minus levoglucosan. For AMS PMF factors, functional group assignments were made by selecting a compound representative of the factor (levoglucosan for BBOA, 2-methyltetrol dimer for Isoprene-OA, $C_8O_4H_{14}$ for LO-OOA, and fulvic acid for MO-OOA) and adjusting the functional groups up or down to result in an overall O:C and H:C more consistent with the PMF factor. Molecular masses were kept below 500 g mol^{-1}. All compound/factor concentrations were set ≥zero and the total mass normalized to reproduce total organic aerosol mass measured by the GT AMS.

AIOMFAC Functional Groups						Number of Functional Groups in Organic Species											
Group Name		Group molecular weight (g/mol)	Number of C in group	Number of O in group	Number of H in group	MO-OOA	BBOA	2-methyltetrol dimer	Isoprene-OA	LO-OOA	2-methyltetrol	Pinic acid	C5-alkene triol	2-methylglyceric acid	levoglucosan	Pinonic acid	Hydroxyglutaric acid
alkyl (standard)	(CH3)	15	1	0	3	0	0	1	2	2	0	2	1	1	0	3	0
	(CH2)	14	1	0	2	2	1	0	1	1	0	2	0	0	0	2	2
	(CH)	13	1	0	1	0	1	0	1	1	0	2	0	0	1	2	0
	(C)	12	1	0	0	0	0	1	1	0	0	1	0	0	0	1	0
alkyl in alcohols	(CH3[alc])	15	1	0	3	0	0	1	0	0	1	0	0	0	0	0	0
	(CH2[alc])	14	1	0	2	0	0	0	0	0	0	0	0	0	0	0	0
	(CH[alc])	13	1	0	1	0	0	0	0	0	0	0	0	0	0	0	0
	(C[alc])	12	1	0	0	0	0	0	0	0	0	0	0	0	0	0	0
alkyl in tail of alcohols	(CH3[alc-tail])	15	1	0	3	0	0	0	0	0	0	0	0	0	0	0	0
	(CH2[alc-tail])	14	1	0	2	0	0	0	0	0	0	0	0	0	0	0	0
	(CH[alc-tail])	13	1	0	1	0	0	0	0	0	0	0	0	0	0	0	0
	(C[alc-tail])	12	1	0	0	0	0	0	0	0	0	0	0	0	0	0	0
alkyl bonded to OH (OH separately)	(CH3 [OH])	15	1	0	3	0	0	0	0	0	0	0	0	0	0	0	0
	(CH2 [OH])	14	1	0	2	1	0	3	1	2	2	0	2	1	0	0	0
	(CH[OH])	13	1	0	1	3	2	2	1	1	1	0	0	0	3	1	1
	(C[OH])	12	1	0	0	4	0	1	0	1	1	0	0	1	0	0	0
alkenyl	(CH2=CH)	27	2	0	3	0	0	0	0	0	0	0	0	0	0	0	0
	(CH=CH)	26	2	0	2	0	0	0	0	0	0	0	0	0	0	0	0
	(CH2=C)	26	2	0	2	0	0	0	0	0	0	0	0	0	0	0	0

	(CH=C)	25	2	0	1	0	0	0	0	0	0	0	0	0	0	0	0
	(C=C)	24	2	0	0	0	0	0	0	0	0	0	0	0	0	0	0
aromatic hydro-carbon	(ACH)	13	1	0	1	0	0	0	0	0	0	0	0	0	0	0	0
	(AC)	12	1	0	0	0	0	0	0	0	0	0	2	0	0	0	0
aromatic carbon-alcohol	(ACOH)	29	1	1	1	0	0	0	0	0	0	0	0	0	0	0	0
hydroxyl	(OH)	17	0	1	1	8	2	6	2	4	4	0	3	2	3	1	1
carboxyl	(COOH)	45	1	2	1	2	0	0	2	0	0	2	0	1	0	1	2
	(HCOOH)	46	1	2	2	0	0	0	0	0	0	0	0	0	0	0	0
ketone	(CH3CO)	43	2	1	3	0	0	0	0	0	0	0	0	0	0	0	0
	(CH2CO)	42	2	1	2	0	0	0	0	0	0	0	0	0	0	0	0
aldehyde	(CHO [aldehyde])	29	1	1	1	0	0	0	0	0	0	0	0	0	0	0	0
ester	(CH3 COO)	59	2	2	3	0	0	0	0	0	0	0	0	0	0	0	0
	(CH2 COO)	58	2	2	2	0	0	0	0	0	0	0	0	0	0	0	0
ether	(CH3O)	31	1	1	3	0	0	0	0	0	0	0	0	0	0	0	0
	(CH2O)	30	1	1	2	1	1	1	1	0	0	0	0	0	1	0	0
	(CHO [ether])	29	1	1	1	1	1	0	0	0	0	0	0	0	1	0	0

Table S2: Properties of AIOMFAC surrogates.

	MO-OOA	BBOA	2-methyltetrol dimer	Isoprene-OA	LO-OOA	2-methyltetrol	Pinic acid	C5-alkene triol	2-methylglyceric acid	Levoglucosan	Pinonic acid	Hydroxyglutaric acid
Molecular weight (g/mol)	414	146	254	250	178	136	186	118	120	162	186	148
O:C	1.00	0.67	0.70	0.70	0.50	0.80	0.44	0.60	1.00	0.83	0.30	1.00
H:C	1.57	1.67	2.20	1.80	2.25	2.40	1.56	2.00	2.00	1.67	1.80	1.60
OM/OC	2.46	2.03	2.12	2.08	1.85	2.27	1.72	1.97	2.50	2.25	1.55	2.47

Table S3: SMILES strings for organic compounds and factors.

Model Species	SMILES representation
MO-OOA	C1(C(C(C(C2C1C(C3(C(O2)(C(C(OC3)(CO)O)O)O)O)=O)O)O)O)(C(=O)O)O
BBOA	C1C2C(CC(C(O1)O2)O)O
Isoprene-OA	C(=O)(O)C(C)C(O)COC(C)(CO)CC(=O)O
LO-OOA	CC(C)CC(O)(CO)C(O)CO
2-methyltetrol (monomer)	C(O)C(O)(C)C(O)CO
Pinic acid	CC1(C(CC1C(=O)O)CC(=O)O)C
C5-alkene triol	C(O)C(C)=C(O)CO
2-methylglyceric acid	CC(CO)(C(=O)O)O
Levoglucosan	C1C2C(C(C(C(O1)O2)O)O)O
Pinonic acid	CC(=O)C1CC(C1(C)C)CC(=O)O
Hydroxyglutaric acid	C(CC(=O)O)C(C(=O)O)O
2-methyltetrol dimer	OCC(O)(C)C(O)COC(CO)(C)C(O)CO

Table S4: Saturation concentrations at T_{ref}=298.15 K and enthalpies of vaporization (ΔH in kJ/mol) for 298.15±7K fitted to reproduce ambient partitioning or predicted based on vapor pressure for the pure species. Fitted values are based on traditional absorptive partitioning to an organic-only medium:

$$F_{p,i} = (1 + T_{ref}/T \times \exp[\Delta H/8314\ kJ^{-1}\ mol\ K \times (1/T_{ref}-1/T)\ 1/K] \times C^* / (M_i \times N)\ )^{-1} \quad (S1)$$

where M_i is the molecular mass of the species and N = C_{org}/200 g mol^{-1}. EVAPORATION, MYN, and NN structure-based estimates are provided by UMANSYSPROP (Topping et al., 2016) available at http://umansysprop.seaes.manchester.ac.uk. Lower and upper bound parameter estimates are provided for the 95% confidence interval of the fits to ambient data. NS indicates the parameter was not statistically significant in the fit. AIOMFAC adjusted C* reflect base values multiplied by 0.238 (Adj Psat sensitivity calculations).

	2-methyltetrol (monomer)	2-methyltetrol dimer	C5-alkene triol	2-methylglyceric acid	pinic acid	pinonic acid	hydroxyglutaric acid	levoglucosan
C* (μg m^{-3})								
SIMPOL[a]	5	6.6E-07	565	4899	7	980	2	16
EVAPORATION[b]	34	2.8E-06	63	301	22	7213	9	18
MYN[c]	507	2.1E-01	7217	2594	1051	18366	152	8172
NN[d]	10	5.8E-08	1205	115	53	4556	1	269
Fit to Ambient	1.8	NA	2.1	2.7	3.5	81	0.2	0.5
Fit to Ambient (lower bound)	1.5	NA	1.7	2.3	3.0	70	0.2	0.4
Fit to Ambient (upper bound)	2.1	NA	2.5	3.2	4.2	94	0.3	0.7
AIOMFAC Adjusted (Adj Psat)	7.7	NA	14	69	5.1	1700	2	4
ΔH^{vap} (kJ mol^{-1})								
SIMPOL	107	167	89	78	99	76	102	98
EVAPORATION	107	176	105	97	112	89	112	115
MYN	92	120	83	86	88	78	95	81
NN	117	211	94	106	108	87	127	103
Fit to Ambient	122	NA	129	71	120	NS	NS	NS
Fit to Ambient (lower bound)	87	NA	84	35	84	NS	NS	NS
Fit to Ambient (upper bound)	158	NA	178	108	158	NS	NS	NS

[a]SIMPOL: Pankow and Asher (2008)
[b]EVAPORATION: Compernolle et al. (2011). Used with AIOMFAC.
[c]MYN: Myrdal and Yalkowsky (1997) vapor pressure method with Nanoolal et al. (2004) boiling point method.
[d]NN: Nannoolal et al. (2008) vapor pressure method with Nanoolal et al. (2004) boiling point method.

Table S5: Average concentrations of particulate ammonium and sulfate and their ratios at the SOAS Centreville site from 1 June 2013 to 15 July 2013.

Instrument	Number of Hourly Aggregated Observations	Mean Ammonium ($\mu g\ m^{-3}$)	Mean Sulfate ($\mu g\ m^{-3}$)	$R_{N/2S}$ Molar Ratio of Means	Mean of Molar Ratio $R_{N/2S}$
GT AMS (Xu et al. 2015a,b) PM_1	881	0.40	1.8	0.59	0.51
CU AMS (Hu et al. 2015) PM_1	646	0.39	2.2	0.47	0.44
SEARCH CTR $PM_{2.5}$	739	0.59	1.8	0.86	0.96
MARGA (Allen et al. 2015) $PM_{2.5}$	948	0.67	2.2	0.81	0.80
URG Corporation Ambient Ion Monitor (AIM) 9000-D $PM_{1\&2.5}$	374	0.91	2.1	1.2	1.4

Table S6: Molar ratio of ammonium to sulfate ($R_{N/S}$) from Silvern et al. (2017) and resulting $R_{N/2S}$.

Dataset	$R_{N/S}$	$R_{N/2S}$
Eastern US CSN Summer 2013 $PM_{2.5}$	1.44	0.72
CU AMS at SOAS CTR PM_1	0.93	0.47
AMS on SEAC^{4}RS aircraft (RMA regression) PM_1	1.21	0.60
SEARCH (five site mean) $PM_{2.5}$	1.62	0.81

Table S7: Average concentration of ammonia at the SOAS Centreville site from 1 June 2013 to 15 July 2013. ppb to $\mu g\ m^{-3}$ conversions assume 303.15 K (1 ppb = 0.68 $\mu g\ m^{-3}$).

Instrument	Number of Hourly Aggregated Observations	Ammonia (ppb)	Ammonia ($\mu g\ m^{-3}$)	Ratio of Means: NH_4^+/NH_x
SEARCH CTR	915	0.38	0.26	0.68
MARGA (Allen et al., 2015)	948	0.75	0.51	0.55
CIMS (You et al., 2014)	799	0.52	0.36	NA
URG Corporation Ambient Ion Monitor (AIM) 9000-D	370	0.85	0.58	0.50

Table S8: Mean C* accounting for the effects of temperature and ideality in CLLPS and EQLB and for pure the species at 298.15 K (Adj Psat, adjusted vapor pressure calculations). For AIOMFAC calculations, C* follows equation 4. Thus, for a system with two liquid phases (α and β) in the particle (PM), the following results:

$$C_i^* = \frac{P_i^{sat}\gamma_i^{\alpha}\left(\sum_k C_k^{PM}\right)}{RT\left(\sum_k {C_k^{\alpha}}/{M_k}\right)}\left(\frac{C_i^{\alpha}}{C_i^{\alpha}+C_i^{\beta}}\right) \quad \text{(S2)}$$

where P_i^{sat} is the pure species vapor pressure at temperature T, γ_i^{α} is the mole-fraction based activity coefficient for species *i* in the α phase, C_i^{α} is the mass concentration of species *i* in the α phase, C_i^{β} is the mass concentration of species *i* in the β phase, M_k is the molecular mass of species *k*, and the summations are over all PM species (water, organic compounds, and inorganic compounds). The C_i^* could be defined analogously for the β phase. For one liquid phase, the equation reduces to:

$$C_i^* = \frac{P_i^{sat}\gamma_i M_{PM}}{RT} \quad \text{(S3)}$$

where the effective PM molecular mass (M_{PM}) is:

$$M_{PM} = \frac{\sum_k C_k^{PM}}{\sum_k {C_k^{PM}}/{M_k}} \quad \text{(S4)}$$

species	CLLPS C* (µg m^{-3})	EQLB C* (µg m^{-3})	Pure Species C* (µg m^{-3})	Ratio EQLB C*/ CLLPS C*	Ratio EQLB C*/ Pure C*
2-methyltetrol	6.0	3.7	7.7	0.62	0.47
pinic acid	13	16	5.1	1.19	3.09
C_5-alkene triol	22	17	14	0.78	1.19
2-methylglyceric acid	43	22	69	0.50	0.31
levoglucosan	1.6	1.4	4	0.90	0.35
pinonic acid	2.0E+04	3.1E+04	1.7E+03	1.55	18.7
hydroxyglutaric acid	0.85	0.60	2	0.71	0.29

Table S9: Mean activity coefficients predicted by AIOMFAC (mole-fraction based) for semivolatile organics (Adj Psat calculations). The β phase was organic-rich in both CLLPS and EQLB calculations.

species	γ CLLPS β phase	γ EQLB β phase	γ EQLB α phase	Ratio: γ_β EQLB/γ_β CLLPS
2-methyltetrol	0.63	0.77	4.7E+03	1.23
pinic acid	5.22	16.21	1.4E+09	3.10
C_5-alkene triol	1.37	2.04	9.3E+04	1.49
2-methylglyceric acid	0.49	0.48	23	0.97
levoglucosan	0.42	1.02	1.4E+05	2.45
pinonic acid	26.60	121.31	1.3E+10	4.56
hydroxyglutaric acid	0.36	0.96	290	2.63

Table S10: Comparison of CMAQ predicted aerosol species concentrations and CSN and SEARCH network observations in the Southeast U.S. NOAA climate region.

Species	Network	n	Mean O_i	Mean O_i	Mean M_i	Mean M_i	Mean(M_i)/ Mean(O_i)	r^2	MB	ME	NMB	NME	FB	FE	IofA	RMSE
		-	µg/ m^3	µmol/ m^3	µg/ m^3	µmol/ m^3	-		µg/ m^3	µg/ m^3	%	%	%	%	-	µg/ m^3
SO_4^{-2}	CSN	225	1.53	0.0159	1.61	0.0168	1.1	0.48	0.07	0.46	5	30	4	32	0.82	0.59
SO_4^{-2}	SEARCH	97	1.77	0.0184	1.56	0.0163	0.9	0.49	-0.22	0.50	-12	28	-16	35	0.82	0.65
NH_4^+	CSN	225	0.27	0.0147	0.40	0.0221	1.5	0.50	0.13	0.20	50	75	44	75	0.79	0.26
NH_4^+	SEARCH	95	0.54	0.0301	0.38	0.0211	0.7	0.37	-0.16	0.26	-30	47	-57	71	0.71	0.31
Na^+	CSN	224	0.05	0.0024	0.12	0.0053	2.3	0.36	0.07	0.08	126	146	72	88	0.62	0.12
Na^+	SEARCH	93	0.05	0.0020	0.12	0.0053	2.6	0.49	0.08	0.08	164	168	74	80	0.49	0.14
Ca^{+2}	CSN	224	0.03	0.0007	0.13	0.0032	4.8	0.45	0.10	0.11	378	387	118	122	0.36	0.17
Ca^{+2}	SEARCH	201	0.03	0.0007	0.16	0.0040	5.9	0.67	0.13	0.13	490	490	118	118	0.23	0.24
Mg^{+2}	CSN	224	0.00	0.0002	0.02	0.0007	4.1	0.04	0.01	0.02	308	379	76	101	0.38	0.02
K^+	CSN	224	0.06	0.0016	0.17	0.0042	2.7	0.09	0.10	0.12	166	186	57	77	0.24	0.18
K^+	SEARCH	201	0.06	0.0015	0.18	0.0045	3.1	0.05	0.12	0.13	207	228	61	78	0.18	0.22
Cl^-	CSN	224	0.02	0.0005	0.03	0.0009	1.6	0.09	0.01	0.03	61	148	17	97	0.46	0.07
NO_3^-	CSN	225	0.31	0.0049	0.27	0.0044	0.9	0.07	-0.04	0.22	-12	73	-48	84	0.43	0.35
NO_3^-	SEARCH	97	0.05	0.0008	0.30	0.0048	6.0	0.18	0.25	0.27	497	542	48	110	0.10	0.62

For a given set of n model predictions, $\{M_i\}$, and observations, $\{O_i\}$:

MB, Mean bias = $\frac{1}{n}\sum_1^n (M_i - O_i)$

ME, Mean error = $\frac{1}{n}\sum_1^n |M_i - O_i|$

NMB, Normalized mean bias = $\frac{\sum_1^n (M_i - O_i)}{\sum_i^n O_i} \times 100\%$

NME, Normalized mean error = $\frac{\sum_1^n |M_i - O_i|}{\sum_i^n O_i} \times 100\%$

FB, Fractional bias = $\frac{1}{n}\frac{\sum_1^n (M_i - O_i)}{\sum_i^n (M_i + O_i)/2} \times 100\%$

FE, Fractional error = $\frac{1}{n}\frac{\sum_1^n |M_i - O_i|}{\sum_i^n (M_i + O_i)/2} \times 100\%$

IofA, Index of agreement = $1 - \frac{\sum_1^n (O_i - M_i)^2}{\sum_i^n (|M_i - \bar{O}| + |O_i - \bar{O}|)^2}$

RMSE, Root mean square error = $\sqrt{\frac{\sum_1^n (M_i - O_i)^2}{n}}$

Figure S1: Observed (CSN, IMPROVE) and modeled (CMAQ) ions for June 1, 2013 to July 15, 2013.

Major cations and anions for the Southeast U.S. NOAA Climate Region (FL, GA, SC, NA, VA)

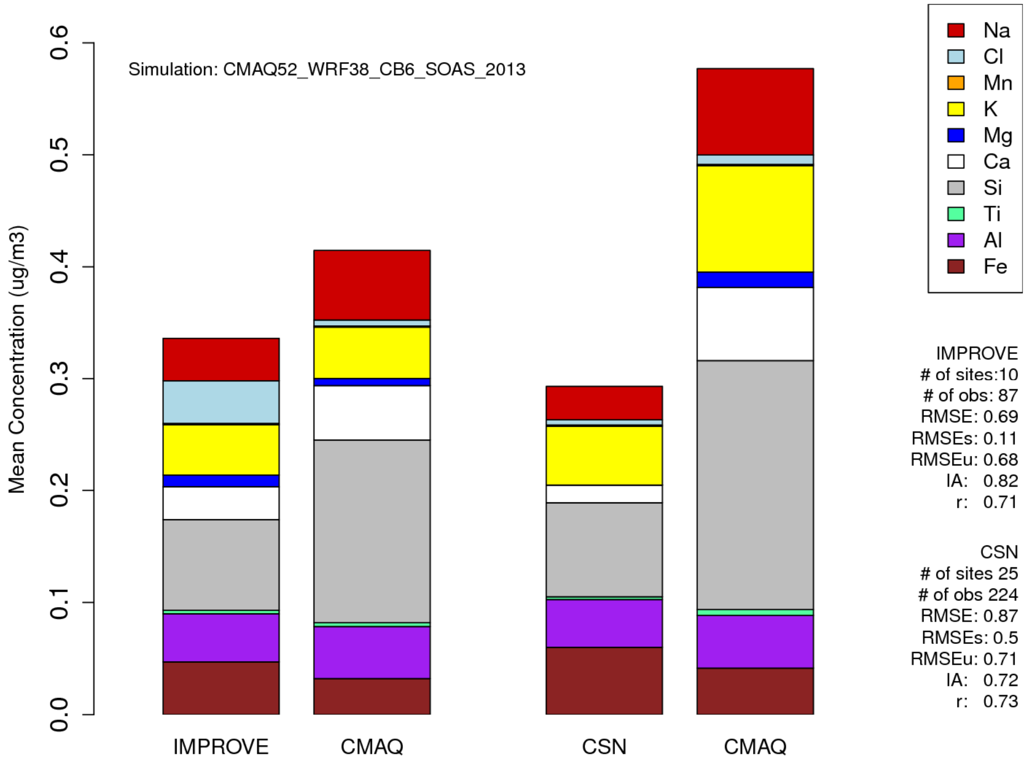

Figure S2: Observed (CSN-circle, SEARCH-triangle) and modeled (CMAQ) ammonium for June 1, 2013 to July 15, 2013. Ammonium is not measured by the IMPROVE network.

(a) Observed Ammonium (μg m^{-3})

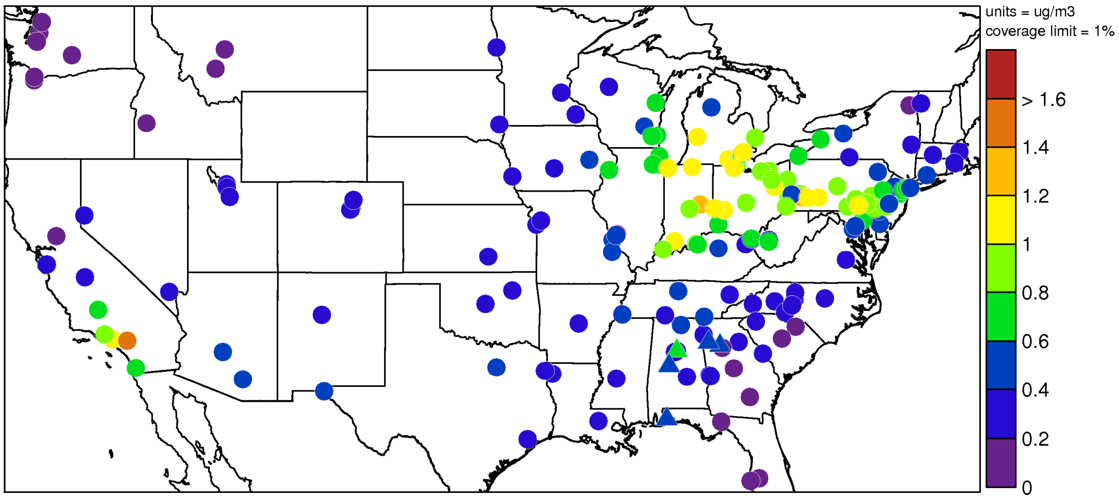

(b) Modeled – Observed Ammonium (μg m^{-3})

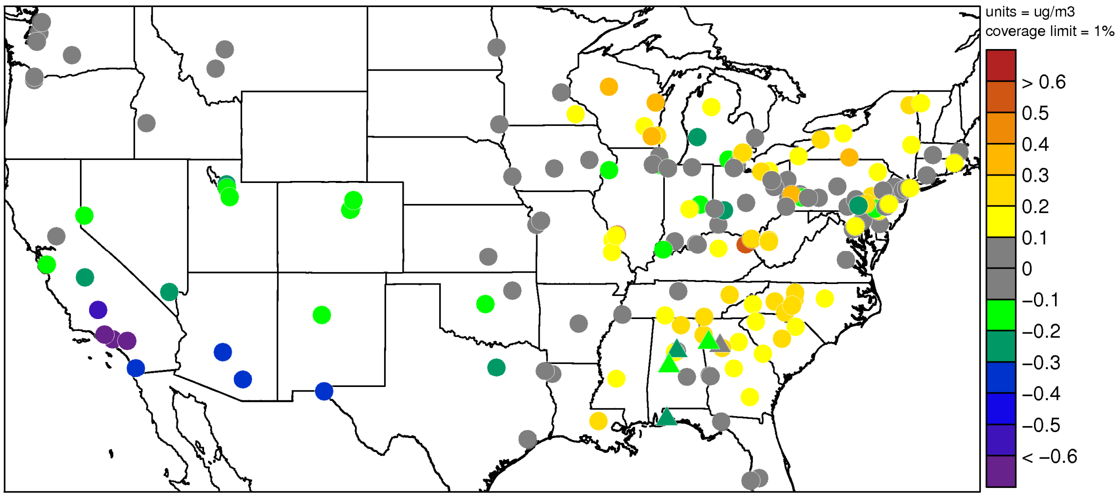

Figure S3: Observed (IMPROVE-square, CSN-circle, SEARCH-triangle) and modeled (CMAQ) sulfate for June 1, 2013 to July 15, 2013.

(a) Observed sulfate (μg m^{-3})

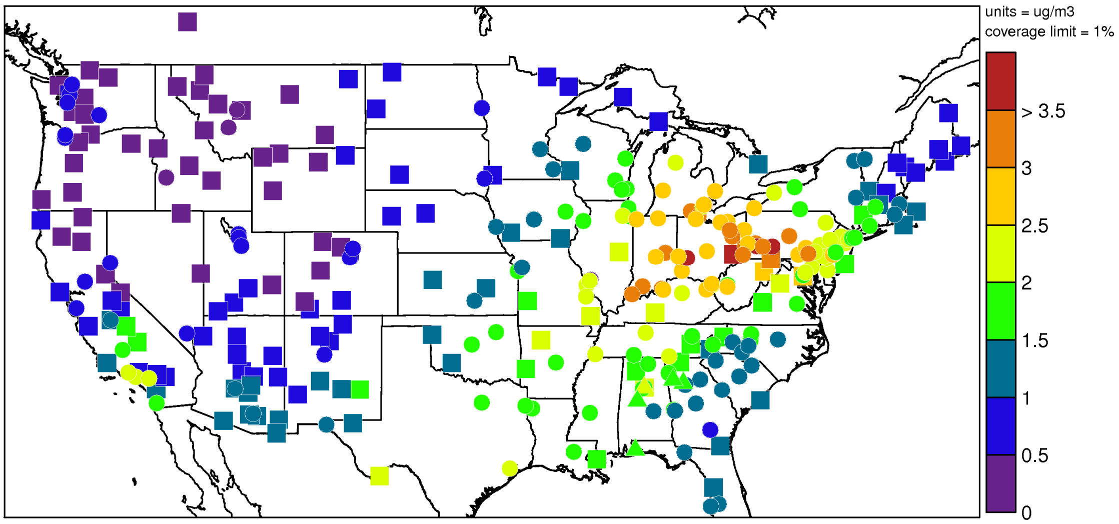

(b) Modeled – Observed sulfate (μg m^{-3})

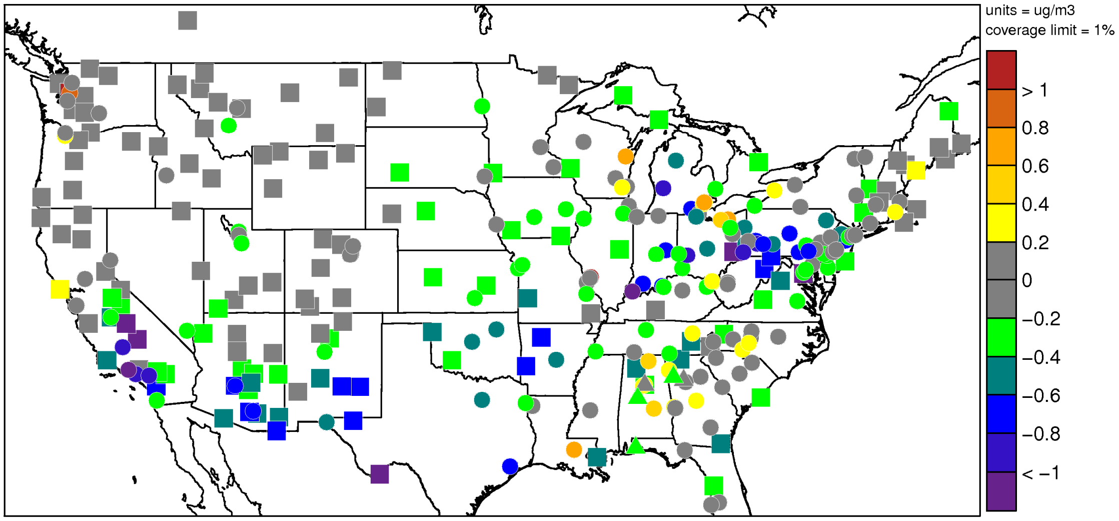

Figure S4: Modeled vs Observed (CSN) Molar Ratio of (a) ammonium to 2 × sulfate and (b) cations to anions (2 × calcium + potassium + sodium + ammonium + 2 × magnesium)/(2 × sulfate + nitrate + chloride).

(a) Ratio of ammonium to 2×sulfate

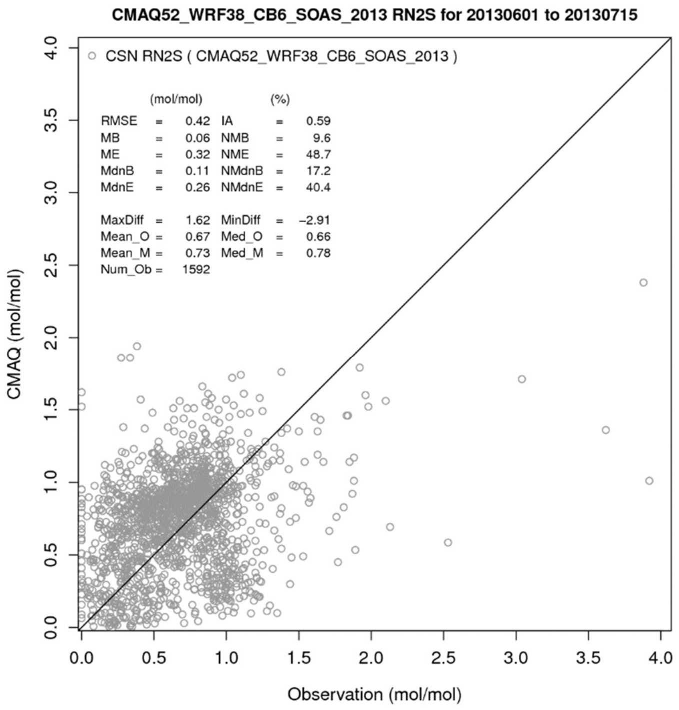

(b) Ratio of cations to anions

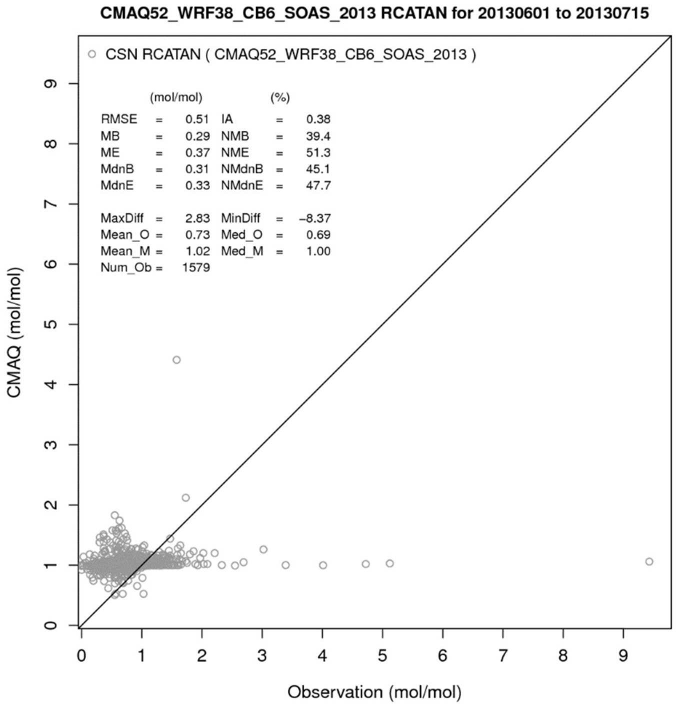

Figure S5: (a) Observed (Ammonia monitoring Network, AMoN), (b) CMAQ simulated, and (c) model bias in gas-phase ammonia concentrations June 1, 2013- July 15, 2013.

(a) AMoN Ammonia (ppb)

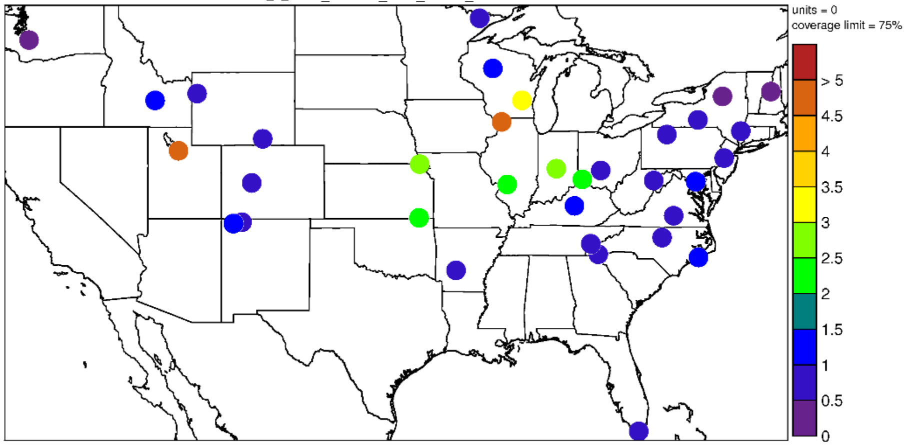

(b) CMAQ Predicted Ammonia (ppb)

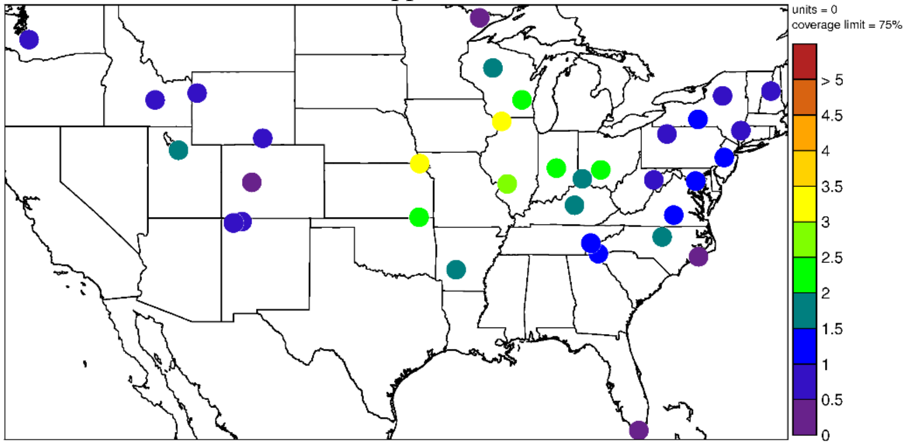

(c) Modeled - Observed Ammonia (ppb)

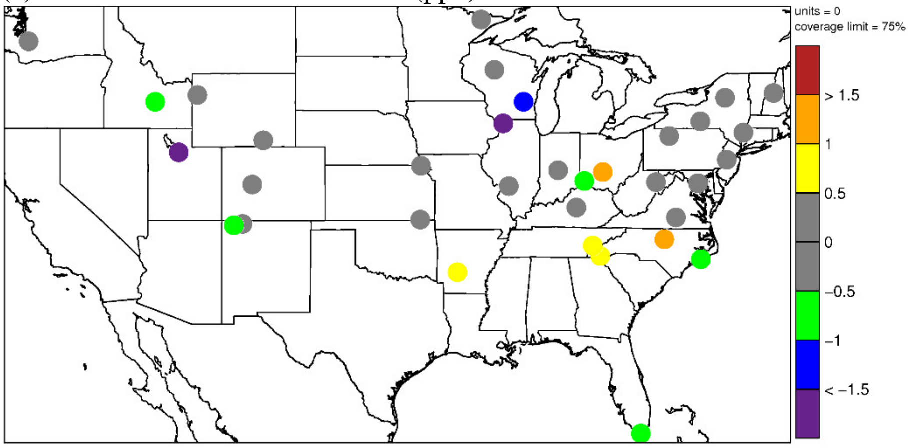

Figure S6: Observed and CMAQ predicted inorganic species at SOAS Centreville site.

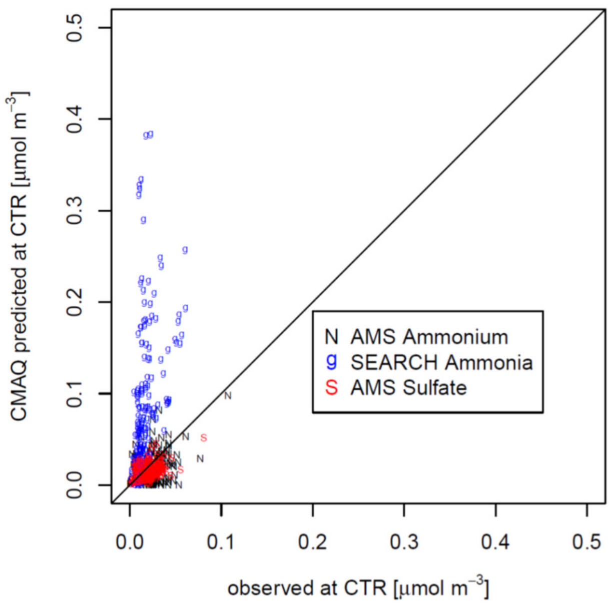

Figure S7: Liquid-liquid phase separation as a function of hour of day predicted by AIOMFAC for the ammonium-sodium-sulfate-nitrate-chloride and organic surrogates system. Shown is the percentage of the time a phase separation was predicted in a certain hour-of-day bin. For reference, the oxygen-to-carbon ratio based separation relative humidity (SRH) as parameterized by You et al. (2013) is shown in blue.

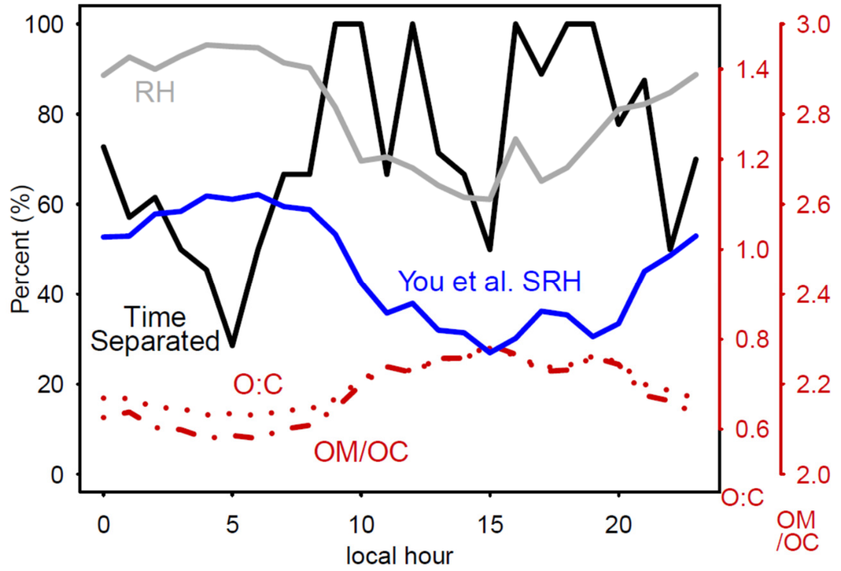

Figure S8: r^2 (square of Pearson's r) between model predicted and observed F_p for each explicit semivolatile species. The x-axis location is arbitrary for the Traditional regression (equation S1). r^2 does not exceed 0.25 for any species or method.

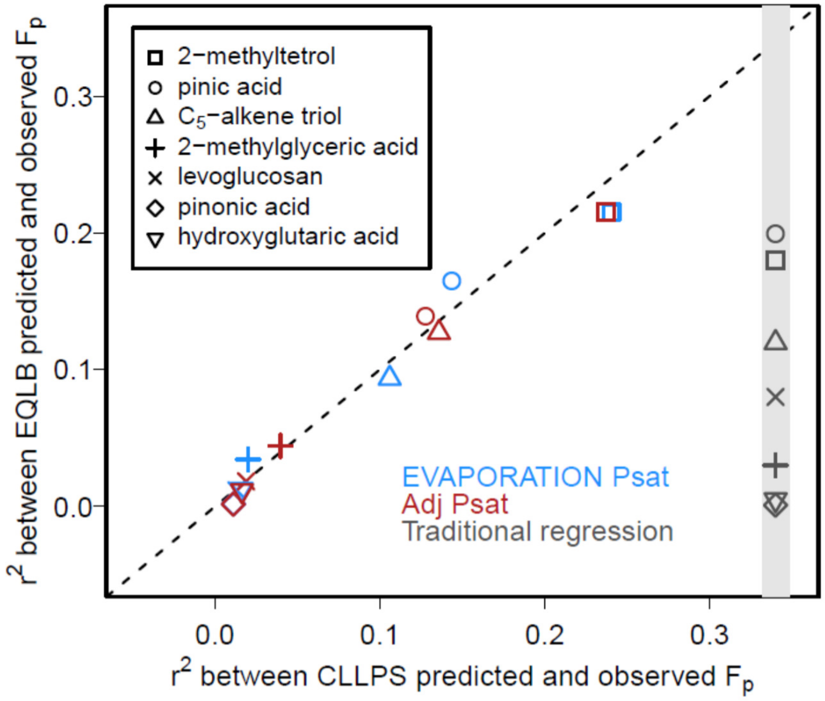

References

Allen, H. M., Draper, D. C., Ayres, B. R., Ault, A., Bondy, A., Takahama, S., Modini, R. L., Baumann, K., Edgerton, E., Knote, C., Laskin, A., Wang, B., and Fry, J. L.: Influence of crustal dust and sea spray supermicron particle concentrations and acidity on inorganic NO_3^- aerosol during the 2013 Southern Oxidant and Aerosol Study, Atmos. Chem. Phys., 15, 10669-10685, doi: 10.5194/acp-15-10669-2015, 2015.

Compernolle, S., Ceulemans, K., and Müller, J. F.: EVAPORATION: a new vapour pressure estimation methodfor organic molecules including non-additivity and intramolecular interactions, Atmos. Chem. Phys., 11, 9431-9450, doi: 10.5194/acp-11-9431-2011, 2011.

Hu, W. W., Campuzano-Jost, P., Palm, B. B., Day, D. A., Ortega, A. M., Hayes, P. L., Krechmer, J. E., Chen, Q., Kuwata, M., Liu, Y. J., de Sá, S. S., McKinney, K., Martin, S. T., Hu, M., Budisulistiorini, S. H., Riva, M., Surratt, J. D., St. Clair, J. M., Isaacman-Van Wertz, G., Yee, L. D., Goldstein, A. H., Carbone, S., Brito, J., Artaxo, P., de Gouw, J. A., Koss, A., Wisthaler, A., Mikoviny, T., Karl, T., Kaser, L., Jud, W., Hansel, A., Docherty, K. S., Alexander, M. L., Robinson, N. H., Coe, H., Allan, J. D., Canagaratna, M. R., Paulot, F., and Jimenez, J. L.: Characterization of a real-time tracer for isoprene epoxydiols-derived secondary organic aerosol (IEPOX-SOA) from aerosol mass spectrometer measurements, Atmos. Chem. Phys., 15, 11807-11833, doi: 10.5194/acp-15-11807-2015, 2015.

Myrdal, P. B., and Yalkowsky, S. H.: Estimating pure component vapor pressures of complex organic molecules, Ind. Eng. Chem. Res., 36, 2494-2499, doi: 10.1021/ie950242l, 1997.

Nannoolal, Y., Rarey, J., Ramjugernath, D., and Cordes, W.: Estimation of pure component properties: Part 1. Estimation of the normal boiling point of non-electrolyte organic compounds via group contributions and group interactions, Fluid Phase Equilibr., 226, 45-63, doi: 10.1016/j.fluid.2004.09.001, 2004.

Nannoolal, Y., Rarey, J., and Ramjugernath, D.: Estimation of pure component properties: Part 3. Estimation of the vapor pressure of non-electrolyte organic compounds via group contributions and group interactions, Fluid Phase Equilibr., 269, 117-133, doi: 10.1016/j.fluid.2008.04.020, 2008.

Pankow, J. F., and Asher, W. E.: SIMPOL.1: a simple group contribution method for predicting vapor pressures and enthalpies of vaporization of multifunctional organic compounds, Atmos. Chem. Phys., 8, 2773-2796, doi: 10.5194/acp-8-2773-2008, 2008.

Silvern, R. F., Jacob, D. J., Kim, P. S., Marais, E. A., Turner, J. R., Campuzano-Jost, P., and Jimenez, J. L.: Inconsistency of ammonium–sulfate aerosol ratios with thermodynamic models in the eastern US: a possible role of organic aerosol, Atmos. Chem. Phys., 17, 5107-5118, doi: 10.5194/acp-17-5107-2017, 2017.

Topping, D., Barley, M., Bane, M. K., Higham, N., Aumont, B., Dingle, N., and McFiggans, G.: UManSysProp v1.0: an online and open-source facility for molecular property prediction and atmospheric aerosol calculations, Geosci. Model Dev., 9, 899-914, doi: 10.5194/gmd-9-899-2016, 2016.

Xu, L., Guo, H., Boyd, C. M., Klein, M., Bougiatioti, A., Cerully, K. M., Hite, J. R., Isaacman-VanWertz, G., Kreisberg, N. M., Knote, C., Olson, K., Koss, A., Goldstein, A. H., Hering, S. V., de Gouw, J., Baumann, K., Lee, S.-H., Nenes, A., Weber, R. J., and Ng, N. L.: Effects of anthropogenic emissions on aerosol formation from isoprene and monoterpenes in the southeastern United States, P. Natl. Acad. Sci. USA, 112, 37-42, doi: 10.1073/pnas.1417609112, 2015a.

Xu, L., Suresh, S., Guo, H., Weber, R. J., and Ng, N. L.: Aerosol characterization over the southeastern United States using high-resolution aerosol mass spectrometry: spatial and seasonal variation of aerosol composition and sources with a focus on organic nitrates, Atmos. Chem. Phys., 15, 7307–7336, doi:10.5194/acp-15-7307-2015, 2015b.

You, Y., Renbaum-Wolff, L., and Bertram, A. K.: Liquid-liquid phase separation in particles containing organics mixed with ammonium sulfate, ammonium bisulfate, ammonium nitrate or sodium chloride, Atmos. Chem. Phys., 13, 11723-11734, doi: 10.5194/acp-13-11723-2013, 2013.

You, Y., Kanawade, V. P., de Gouw, J. A., Guenther, A. B., Madronich, S., Sierra-Hernández, M. R., Lawler, M., Smith, J. N., Takahama, S., Ruggeri, G., Koss, A., Olson, K., Baumann, K., Weber, R. J., Nenes, A., Guo, H., Edgerton, E. S., Porcelli, L., Brune, W. H., Goldstein, A. H., and Lee, S. H.: Atmospheric amines and ammonia measured with a chemical ionization mass spectrometer (CIMS), Atmos. Chem. Phys., 14, 12181-12194, doi: 10.5194/acp-14-12181-2014, 2014.

Zuend, A., and Seinfeld, J. H.: Modeling the gas-particle partitioning of secondary organic aerosol: the importance of liquid-liquid phase separation, Atmos. Chem. Phys., 12, 3857-3882, doi: 10.5194/acp-12-3857-2012, 2012.
